# Sphingosine-1-phosphate promotes the differentiation of adipose-derived stem cells into endothelial nitric oxide synthase (eNOS) expressing endothelial-like cells

**DOI:** 10.1186/1423-0127-21-55

**Published:** 2014-06-04

**Authors:** Divya Arya, Shaohua Chang, Paul DiMuzio, Jeffrey Carpenter, Thomas N Tulenko

**Affiliations:** 1 Department of Surgery, Cooper University Hospital and Cooper Medical School of Rowan University, 3 Cooper Plaza, Camden, NJ 08103, USA; 2 Department of Surgery, Thomas Jefferson University College of Medicine, 1025 College Bldg, Philadelphia, PA 19017, USA

## Abstract

**Background:**

Adipose tissue provides a readily available source of autologous stem cells. Adipose-derived stem cells (ASCs) have been proposed as a source for endothelial cell substitutes for lining the luminal surface of tissue engineered bypass grafts. Endothelial nitric oxide synthase (eNOS) is a key protein in endothelial cell function. Currently, endothelial differentiation from ASCs is limited by poor eNOS expression. The goal of this study was to investigate the role of three molecules, sphingosine-1-phosphate (S1P), bradykinin, and prostaglandin-E1 (PGE1) in ASC endothelial differentiation. Endothelial differentiation markers (CD31, vWF and eNOS) were used to evaluate the level of ASCs differentiation capability.

**Results:**

ASCs demonstrated differentiation capability toward to adipose, osteocyte and endothelial like cell phenotypes. Bradykinin, S1P and PGE were used to promote differentiation of ASCs to an endothelial phenotype. Real-time PCR showed that all three molecules induced significantly greater expression of endothelial differentiation markers CD31, vWF and eNOS than untreated cells. Among the three molecules, S1P showed the highest up-regulation on endothelial differentiation markers. Immunostaining confirmed presence of more eNOS in cells treated with S1P than the other groups. Cell growth measurements by MTT assay, cell counting and EdU DNA incorporation suggest that S1P promotes cell growth during ASCs endothelial differentiation. The S1P1 receptor was expressed in ASC-differentiated endothelial cells and S1P induced up-regulation of PI3K.

**Conclusions:**

S1P up-regulates endothelial cell markers including eNOS in ASCs differentiated to endothelial like cells. This up-regulation appears to be mediated by the up-regulation of PI3K via S1P1 receptor. ASCs treated with S1P offer promising use as endothelial cell substitutes for tissue engineered vascular grafts and vascular networks.

## Background

Adult stem cells, such as endothelial progenitor cells [1,2] and bone-marrow derived mesenchymal cells [3,4] have been evaluated for lining the luminal surface of tissue engineered bypass grafts for cardiovascular therapy. The widespread use of these cells in vascular grafts is limited by harvesting difficulties and decreased availability with advancing age and co-morbidities [5,6]. Adipose tissue provides a source of autologous stem cells in large quantities through minimally invasive procedures [7-11] and isolation efficiency is not affected by the gender, advanced age, obesity, renal failure, or vascular disease [12]. When grown in medium with endothelial cell growth supplement, human adipose-derived stem cells (ASCs) express endothelial specific markers such as platelet-endothelial cell adhesion molecule (PCAM-1 or CD31) and von Willebrand’s Factor (vWF). However, the expression of endothelial nitric oxide synthase (eNOS) is limited [11,13-15]. eNOS is a key signaling protein that promotes vascular smooth muscle relaxation, reduces platelet aggregation and provides atheroprotection through the production of nitric oxide [16]. The presence of eNOS is thus a key marker of endothelial cell function [17]. Though prior studies have demonstrated that shear force [11,13], specialized alloys with nanostructures [18], polycaporlactone scaffolds [19], and transfection with adenovirus [10] promote the expression of eNOS in endothelial cells differentiated from ASCs, their use in humans raises practical concerns of biocompatibility, cost, and safety. A simple and practical method of promoting eNOS expression using biologic molecules that are naturally occurring yet easily available is lacking.

This study investigated whether the naturally occurring molecules, sphingosine-1-phosphate (S1P), bradykinin, and prostaglandin-E1 (PGE1) can promote the differentiation of functional eNOS competent endothelial cells from human ASCs. S1P, a key member of the sphingolipid group, is a circulating bioactive lipid metabolite that can trigger a wide variety of biological effects, including cell differentiation, survival, and angiogenesis [20]. Platelet derived S1P has been identified as an activator of eNOS in bovine cultured vascular endothelial cells through binding to EDG receptors [21,22]. S1P is a sphingolipid that acts on 5 types of G-protein-coupled receptors termed S1P1-S1P5, originally termed EDG receptors [22,23]. Of these, the S1P1, S1P2 and S1P3 receptors are the predominant receptors expressed in mammalian cells [24]. S1P1 is involved in activation of eNOS via phosphoinositide 3-kinase (PI3K)/Akt (protein kinase B)-mediated phosphorylation [22,25]. In addition to activating eNOS as described above, S1P receptors can also activate MAP kinase, extracellular-regulated kinase (ERK), phospholipase C (PLC), small guanosine triphosphatase (Rac), protein kinase C (PKC), adenylate cyclase (cAMP) and Ras homologous proteins (Rho) as well as increase intracellular free calcium [26]. The cell type-specific expression of S1P receptors results in a highly complex master regulatory role of S1P. In normal human lung microvascular endothelial cells, bradykinin activates bradykinin B2 receptors that results in activation of eNOS [27-29]. The PGE1 analogue, alprostadil, has been shown to increase eNOS production in human umbilical vein endothelial cells [30,31].

Based on the above reports that S1P, bradykinin, and PGE1 promote the expression of eNOS in native endothelial cells, we hypothesized that S1P, bradykinin, or PGE1 would up-regulate the expression of endothelial cell differentiation markers (CD31, eNOS, and vWF) in endothelial cells differentiated from ASCs. Human ASCs were cultured in endothelial growth medium and exposed to S1P, bradykinin, or PGE1. The effect of S1P, bradykinin, and PGE1 on expression of eNOS, CD31, and vWF was determined by quantitative, real-time reverse transcription polymerase chain reaction (RT-PCR) and confocal microscopy images of immunostaining. In addition, the effect of S1P on cell growth and PI3K were also investigated to provide a better understanding of the underlying mechanism of S1P stimulation on ASC differentiation.

## Methods

### ASCs isolation and cell culture

ASCs were isolated from human abdominal liposuction aspirate as approved by the Cooper University Hospital Institutional Review Board Committee. Briefly, the adipose tissue from liposuction aspirate was rinsed with PBS buffer and incubated with 0.1% collagenase (Northington) for 1 hour at 37°C with vigorous shaking. After 5 minutes centrifugation (1000 × g), the top lipid layer was removed and the remaining liquid portion was centrifuged again to collect the residual cell pellet. The cell pellet was treated with 1 ml water for 10 seconds to lyse the red blood cells followed by a 3^rd^ centrifugation with 10 ml medium. The cell pellet was then plated into T75 cell culture flask with M-199 containing 10% FBS and placed into a humidified tissue culture incubator. The next day, the floating cells were removed by medium change and the adherent ASCs were grown to confluence as passage zero cells. Passage 1 or passage 2 cells were used in this study to test the effect of S1P (Echelon), bradykinin (Sigma) and PGE (Sigma) on ASCs endothelial differentiation.

### ASCs multipotency test

ASCs were cultured in different media based on experimental design. M-199 was used as non-differentiating maintenance medium. Adipogenic and osteogenic differentiation media were purchased from Invitrogen and endothelial differentiation medium (EGM2) was from Lonza. Two specific staining methods were used to evaluate adipogenic and osteogenic differentiation, Oil red O (Sigma) for fat and Alizarin red (Sigma) for calcium deposit. Endothelial differentiation was measured by CD31 expression using flow cytometry. 5 × 10^5^ cells were prepared for 3 cell lines: un-differentiated ASCs, ASCs differentiated endothelial like cells or HUVECs. Then the cells were incubated with anti-CD31-FITC (BD Biosciences) for 30 minutes and washed before analyzed by Accuri C6 flow cytometer (BD Biosciences).

### RNA isolation and real-time polymerase chain reaction (PCR)

Total RNA was isolated from cultured cells using Trizol reagent (Life Technologies) according to the manufacturer’s RNA isolation protocol. Reverse Transcription was performed to transcribe and amplify mRNA into cDNA using the SuperScript^TM^ II Reverse Transcriptase kit (Life Technologies). Real-Time PCR (7500 fast, Applied Biosystem) was used to measure the expression of eNOS, CD31, and vWF at mRNA level. GAPDH was used to normalize the expression of the target genes.

### Immunostaining and confocal microscopy

Cultured cells were fixed with 4% formalin for 10 minutes and blocked with 5% BSA solution for 1 hour. Then the cells were incubated with different specific primary antibodies, eNOS (BD Biosciences), S1P1 (Cayman Chemical, Ann Arbor, MI) or PI3K (Abcam, Cambridge, MA) overnight in cold room and the corresponding AlexaFluor 488 or 594 conjugated secondary antibody (Molecular Probe) for 1 hour. The slides were then mounted and viewed under an Olympus confocal microscope and images were captured using Fluoview FV100 software.

### Cell growth measured by MTT assay, cell counting and EdU DNA-incorporation assay

ASCs were cultured in the presence or absence of S1P. For MTT assay, cell culture was incubated with MTT solution (Sigma-Aldrich) at 0.5 mg/ml for 2 hours at 37°C. The formazan product was dissolved in 200 μl DMSO then measured at 570 nm for absorbance using SpectroMax microplate reader (Molecular device). Cell counting was measured by Coulter counter (Beckman) after cells were dissociated by trypsin. Lastly, EdU (10 μM) was added to cultured cells for 2 days incubation. Cells were fixed with 4% formalin. EdU incorporated into the nucleus was detected with azide conjugated with Alexa Fluor™ 488 according to the manufacturer’s instruction (Click-iT™ EdU cell proliferation kit, Invitrogen). All nuclei were counted with the aid of DAPI staining.

### Western blotting

Protein samples from cultured cells were isolated by using extraction buffer [50 mM Tris (pH 6.8), 20% glycerol, 1% SDS, and a protease inhibitors cocktail (Sigma, St. Louis, MO)]. The protein concentration was determined using the DC protein assay kit (Bio-Rad, Hercules, CA). 40 μg total protein from each sample was loaded to 4 – 20% precast gels for two hours running at 120 V and then transferred to PVDF membranes overnight at 24 at 4°C. Membrane were blocked with 5% bovine serum albumin blocking buffer for 1 hr. Primary antibody anti-PI3K (Abcam), anti-S1P1 receptor (Cayman Chemical Company) or anti-β-actin (Sigma) was incubated at 4°C overnight or 2 hours at room temperature then followed by 1-h incubation with corresponding secondary antibodies from Molecular Probe. Target protein bands were visualized and quantitated by scanning with Fluo Chemi Q imager (Protein Simple, Santa Clara, CA).

### Data analysis

Histograms were constructed to compare the relative expression of CD31, eNOS, and vWF in the experimental group (treated with S1P, bradykinin or PGE1) and untreated control. Within the experimental group, statistical comparisons were performed by analysis of variance (ANOVA) and multiple comparisons were done with Tukey’s post hoc tests. Values were expressed as mean ± SE. Differences were considered significant at *p <* .05. Student *t*-test was performed to compare the cell growth and PI3K expression between control and S1P treated cells. Asterisks indicate statistical significance with *p* < .05.

## Results

### ASCs differentiation to adipose, osteocyte and endothelial like cells

**Figure 1:**
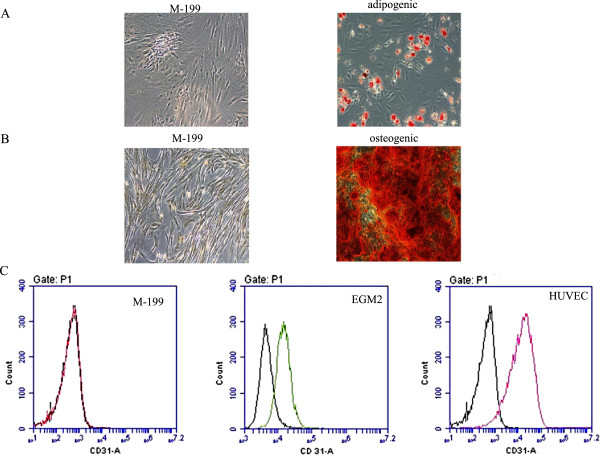
**ASC differentiation to adipose, osteocyte and endothelial like cells.** Oil Red O staining was used to detect lipid droplet formation during adipogenic differentiation **(panel A)**. The left side image depicts control ASCs grown in M-199 medium while the right one shows ASCs grown in adipogenic differentiation medium. Alizarin Red staining was used to measure calcium deposition during osteogenic differentiation **(panel B)**. The left side image depicts control ASCs grown in base medium M-199 while the right one shows ASCs grown in osteogenic differentiation medium. Endothelial differentiation was measured by CD31 flow cytometry **(panel C)**. Left side is undifferentiated ASCs grown in M-199 medium, the middle is differentiated ASCs grown in EGM2 medium and the right is HUVECs endothelial cell control.

ASCs multipotency was determined using three lineage differentiation pathways, including adipogenic, osteogenic and endothelial differentiation. Figure 1 shows each differentiation result. In panel A, ASCs grown in adipogenic differentiation medium display Oil red O staining of lipid droplets while there was no staining in control group. The formation of lipid droplets is a key feature of adipose cells. In panel B, ASCs grown in osteogenic differentiation medium show intensive red staining for calcium deposits while no staining was observed in control cells. Calcium deposition is a key feature of osteocytes. In panel C, there was no CD31 expression in undifferentiated ASCs while ASCs grown with EGM2 expressed endothelial marker CD31 similar to positive HUVECs control.

### Effect of S1P, bradykinin, and PGE1 on ASCs endothelial differentiation

**Figure 2:**
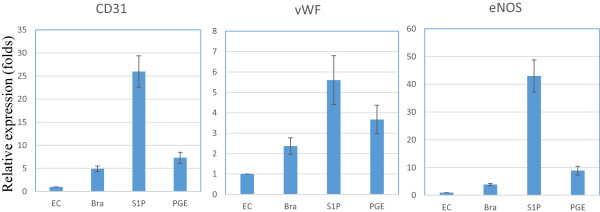
**The effect of bradykinin (Bra), S1P and PGE on the expression of endothelial differentiation markers CD31, vWF and eNOS in ASCs differentiated to endothelial-like cells.** Real-Time PCR was performed to evaluate target gene expression. GAPDH was used to normalize target gene expression levels. A no treatment group was used as control to calculate relative expression between treatments groups. Left panel is for CD31, middle panel is for vWF and right panel is for eNOS. A total n = 6 individual ASC cell lines were tested.

Three molecules were selected to promote ASCs endothelial differentiation and real-time PCR was used to evaluate up-regulation of related genes. Figure 2 shows the relative expression of CD31, vWF and eNOS mRNA in bradykinin, S1P or PGE1 treated groups compared to untreated cells. Significant differences in CD31 expression were noted between the treated and untreated groups (*p <* .01, n = 6). Compared with untreated cells, S1P increased expression of CD31 by 26-fold, bradykinin increased CD31 by 4.9-fold, and PGE1 increased CD31 by 7.3-fold. Post-hoc analysis showed that cells treated with S1P showed significantly greater expression of CD31 than all the other groups (*p <* .05, n = 6). Cells treated with bradykinin, S1P and PGE1 showed similar trend of up-regulation on vWF expression as CD31. However, the increase in vWF was much less than that of CD31. Interestingly, eNOS also showed the similar trend with even a greater change. Compared with untreated cells, S1P increased expression of eNOS by 43-fold, bradykinin increased eNOS expression by 3.9-fold, and PGE1 increased eNOS by 8.9-fold. Post-hoc analysis also indicated that cells treated with S1P had significantly greater expression of eNOS than the others (*p <* .05, n = 6).

### eNOS protein localization by immunostaining and confocal microscopy

**Figure 3:**
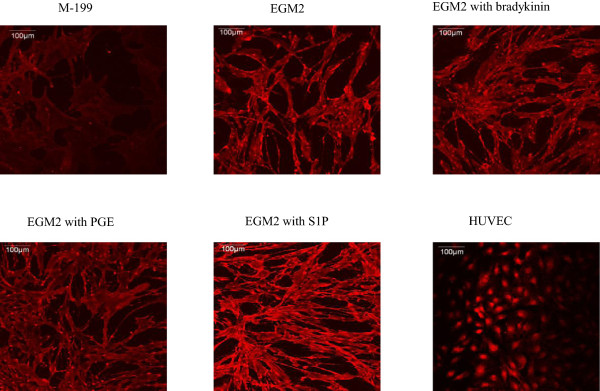
**Immunostaining for eNOS in six different experimental groups.** Cells were grown in M-199, EGM2, EGM2 + bradykinin treatment, EGM2 + PGE treatment, EGM2 + S1P. Red fluorescent staining shows the localization of eNOS. ASCs grown in M-199 served as an undifferentiated negative control and HUVEC served as positive controls. Cells treated with S1P show greater red fluorescence intensity compared to the other groups.

Figure 3 showed a direct visual conformation of eNOS distribution in ASCs differentiated to endothelial like cells using confocal microscopy. Undifferentiated ASCs were used as negative control and the endothelial cell line HUVEC was used as a positive control. Images of immunostaining demonstrated that S1P promoted greater expression of eNOS protein (red fluorescent staining) than the other groups with the exception of the positive control HUVEC. This result was consistent with PCR data and further supported S1P promoting ASCs differentiation to an endothelial cell phenotype. Analysis of cellular morphology showed that treatment with S1P, bradykinin, or PGE1 resulted in a more organized cell structure. Morphologically, untreated cells are long, spindle-like, and widely dispersed. The treated cells have a more organized cell structure with segregated cell-type partitioning; however their morphology is not similar to that of HUVEC controls.

### Effect of S1P on cell growth during ASCs endothelial differentiation

**Figure 4:**
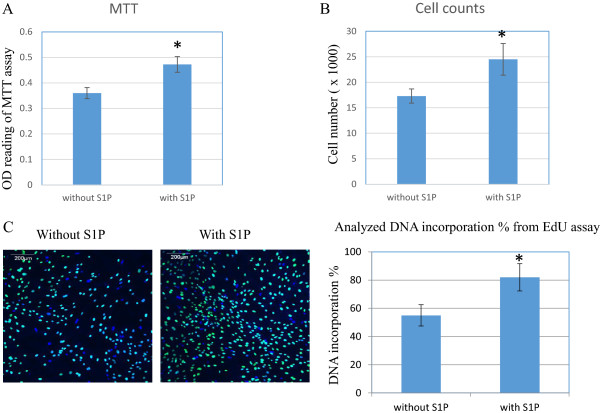
**The effect of S1P on cell growth during differentiation of ASCs to the endothelial phenotype.** Cell growth was evaluated by MTT assay **(panel A)**, direct cell count **(panel B)** and EdU DNA-incorporation kit **(panel C)**. Green staining indicates newly synthesized DNA with EdU incorporation and blue staining is DAPI staining to highlight nuclei. Four individual ASCs cell lines were used. Each cell line was subcultured into two identical sets, one for S1P treatment and one without S1P for control. Student *t*-test was performed to compare the difference between control and S1P treated cells. *indicated statistical significance with *p* < .05.

Both PCR and immunostaining results demonstrate that S1P promotes ASCs differentiation to an endothelial cell phenotype. In addition to the effect of S1P on ASC differentiation, we also evaluated the effect of S1P on cell growth and proliferation. In this study, multiple methods were used to evaluate cell growth (Figure 4). Panel A showed MTT assay, panel B was from direct cell count and panel C measured DNA incorporation during cell growth using EdU staining to distinguish newly synthesized DNA. All measurement clearly demonstrated a significant increase on cell viability (panel A), cell growth (panel B) and cell proliferation (panel C) in S1P treated cells. Student *t*-test showed statistical significance with *p* < .05, n = 4.

### The expression of S1P1 receptor in ASC-differentiated endothelial cells and the up-regulation of PI3K by S1P

**Figure 5:**
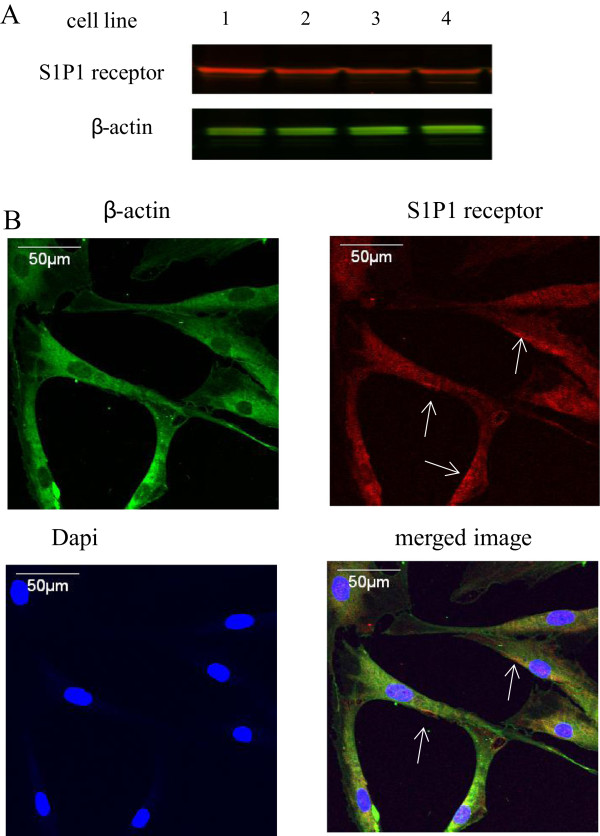
**The expression of S1P1 receptor protein in ASC-differentiated to endothelial-like cells. Panel A**: Western-blot for S1P1 and β-actin in four cell lines of ASC-differentiated endothelial cells. **Panel B**: Immunostaining for S1P1 and β-actin in ASC-differentiated to endothelial cells. Red fluorescence is for S1P1, green fluorescence is for β-actin, blue fluorescence is DAPI for nuclei. White arrows show the membrane distribution of S1P1. Western-blot coupled to immunostaining confirmed the expression of S1P1 in ASC-differentiated to endothelial-like cells.

**Figure 6:**
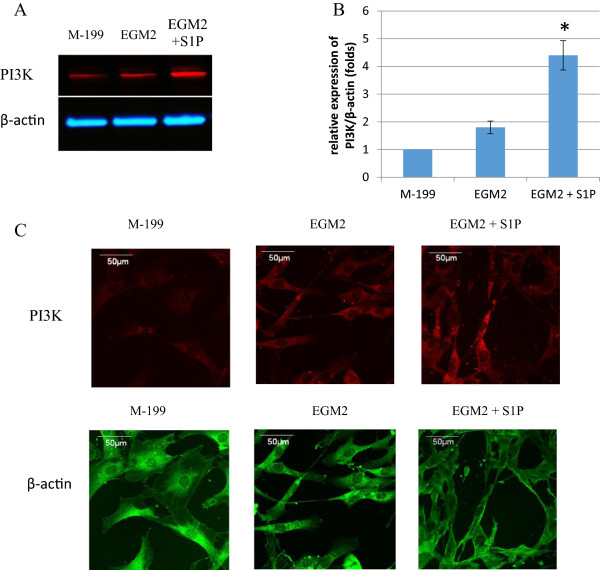
**The up-regulation of PI3K in S1P treated ASC-differentiated endothelial cells.** Panel **A**: Western-blot for PI3K and β-actin in ASCs (M-199), ASC-differentiated endothelial cells (EGM2) and S1P treated ASC-differentiated endothelial cells (EGM2 + S1P). Panel **B**: Analyzed Western-blot data from four ASC cell lines, *indicated statistical significance with *p* < .05. **C**: Immunostaining for PI3K and β-actin in ASC-differentiated endothelial cells. Red fluorescence is for PI3K and green fluorescence is for β-actin. Both Western-blot and immunostaining demonstrate the up-regulation of PI3K by S1P in ASC-differentiated endothelial cells.

After showing the promoting function of S1P on ASC endothelial differentiation and cell growth, our next step was to elucidate underlying mechanism of S1P stimulation. Based on prior studies [25,32], we hypothesized that S1P1 and PI3K were involved in this process. Western-blots of four individual cell lines provided a strong evidence of the existence of S1P1 in ASC-differentiated endothelial cells (Figure 5, panel A). Immunostaining confirmed the expression of S1P1 by positive staining and white arrows indicates membrane localization of S1P1 (Figure 5, panel B). Figure 6 shows that S1P induced the up-regulation of PI3K in ASC-differentiated endothelial cells. The PI3K band is increased in S1P-treated cells (Figure 6, panel A). Analyzed Western-blot data indicated a statistically significant increase of PI3K in S1P treated cells (Figure 6, panel B). Immunostaining showed an increased PI3K staining intensity and the cytoplasmic distribution of PI3K (Figure 6, panel C). Our results suggest that S1P up-regulates PI3K likely via the S1P1 receptor.

## Discussion

This is the first study to investigate the effect of three naturally occurring and easily available biological molecules, S1P, bradykinin, and PGE1, in promoting the differentiation of ASCs into an endothelial like cell phenotype. The main finding of this study is that S1P up-regulates the expression of the endothelial cell differentiation markers, eNOS, CD31 and vWF in endothelial like cells differentiated from ASCs. We cultured ASCs in EGM2 medium, which promotes their differentiation toward an endothelial like cells, and then stimulated the ASCs with S1P, bradykinin, or PGE1. We observed significant differences in mean expression of CD31 and eNOS between the treated and untreated groups. Treatment with S1P resulted in 26-fold increase in expression of CD31, an early marker of endothelial cell differentiation, and 43-fold increase in the expression of eNOS, a marker of more mature differentiation. Confocal microscopy confirmed higher expression of eNOS in cells treated with S1P compared to untreated cells. Cell growth measurements demonstrate that S1P promotes cell proliferation during ASC-to-endothelial differentiation. We also demonstrated the existence of S1P1 receptor in ASC-differentiated endothelial cells and the up-regulation of PI3K by S1P. Taken together, these findings suggest that S1P is a promising molecule that can promote the differentiation of ASC to an eNOS competent endothelial cell, a process which may be driven by up-regulation of PI3K via S1P1 receptor.

In the present study, particular attention was paid to the isolation process of the stem cells. Stem cell multipotency was assessed for each batch of ASCs isolation. In order to demonstrate that the ASCs isolated in this study were indeed multipotent, we showed differentiation into second cell types, namely adipocytes grown in adipogenesis induction medium and osteocytes grown in osteogenesis induction medium. We also set up negative controls of our experiment, i.e. ASCs grown in base medium. The negative control did not form lipid droplets or calcium deposits. They also did not express endothelial markers. Thus, these observations indicate that the increased expression of CD31 and eNOS noted in these experiments by S1P, bradykinin, or PGE1, was the result of differentiation rather than a selection bias.

The mechanism through which S1P promotes differentiation of ASCs into endothelial cells is not known. Prior reports have shown the role of S1P in cell differentiation, e.g., during embryonic development, S1P plays a critical role in stem cell differentiation and apoptosis [33,34]. S1P has been shown to promote the differentiation of stem cells into other types of adult cells such as oligodendroglial lineage [35] and smooth muscle cells [36]. In native endothelial cells, S1P exerts its action by binding to a family of G-protein coupled receptors, called S1P1-S1P5, previously termed as endothelial differentiation gene receptors [21]. S1P induces eNOS activation and endothelial barrier enhancement through PI3K pathway [22,32]. Prior studies also have shown that PI3K pathway plays an important role for endothelial cell differentiation in embryonic stem cells [37,38]. Therefore, we hypothesized that the mechanism of S1P promotion is also through PI3K pathway. We first showed the expression and localization of S1P1 receptor in ASC-differentiated endothelial cells. Then we demonstrated the up-regulation of PI3K by S1P treatment. Our results suggest that the up-regulation of PI3K by S1P is likely mediated by the S1P1 receptor, and PI3K up-regulation is a likely mechanism through which S1P promotes ASC differentiation to an eNOS competent endothelial phenotype. Additional pathways may be involved, but future studies will be required to address this question.

Of the three molecules, S1P appears to have the greatest potential of promoting expression of markers in endothelial cells differentiated from ASCs. Though bradykinin and PGE1 both increased the expression of CD31, eNOS and vWF, marker expression for bradykinin and PGE1 treated cells was not as high as S1P group. Prior studies have reported that PGE1 and bradykinin promote the expression of eNOS in natural endothelial cells and the pathways through which such activation occurs has been described [27,31]. The observed less difference in eNOS expression in the bradykinin and PGE1 groups in our study may be due to the relatively small sample size. Alternatively, it is possible that endothelial cells differentiated from ASCs do not have the same response to bradykinin and PGE1 as native endothelial cells. Future studies will be required to determine if bradykinin and PGE1 have a supplemental role or a synergistic effect with S1P in promoting endothelial cell differentiation.

Our findings that S1P promotes differentiation of ASCs to endothelial cells have considerable implications for future clinical uses. S1P is a polar, bioactive sphingolipid which can be detected and quantified at nM concentrations [39]. Given easy accessibility, ASCs incubated with S1P could offer a ready source of EC-like cells for developing tissue engineered bypass grafts for cardiovascular therapy and/or neovascularization therapy in ischemic tissues. Further refinement of techniques, including the use of a combination of molecules and/or biophysical methods will be required to achieve optimal differentiation of ASCs into functional endothelial cells.

## Conclusion

In summary, this study provides new information regarding the use of S1P for stimulating the differentiation of human ASCs to endothelial-like cells. When cultured in endothelial growth supplement and exposed to stimulating effect of S1P, human ASCs showed markedly increased eNOS expression. S1P is a naturally occurring and easily available biological molecule. Endothelial-like cells differentiated from ASCs and treated with S1P offer promising use as endothelial cell substitutes for tissue engineered vascular grafts and neovascularization.

## Abbreviations

ASCs: Adipose-derived stem cells; CD31: Platelet-endothelial cell adhesion molecule; eNOS: Endothelial nitric oxide synthase; EGM2: Endothelial differentiation medium; HUVECs: Human umbilical vein endothelial cells; PGE1: Prostaglandin-E1; S1P: Sphingosine-1-phosphate; vWF: Von willebrand’s Factor; Bra: Bradykinin.

## Competing interests

The authors declare that they have no competing interests.

## Authors’ contributions

DA carried out the cell culture, ASC differentiation, PCR, data analysis and manuscript preparation. SC carried out Western-blot and reviewed manuscript. PD and JC participated project design, data analysis and manuscript preparation. TT supervised experimental design, data analysis and reviewed manuscript. All authors read and approved the final manuscript.
